# Experimental Study on the Effect of Calcium Aluminate Cement Addition on the Drying and Physical Properties of Refractory Castables Containing Colloidal Silica †

**DOI:** 10.3390/ma17215308

**Published:** 2024-10-31

**Authors:** Antti Piippo, Kyösti Ruotanen, Ville-Valtteri Visuri, Niko Poutiainen, Eetu-Pekka Heikkinen

**Affiliations:** 1 Bet-Ker Oy, Joutsentie 4, 84100 Ylivieska, Finland; kyosti.ruotanen@betker.fi (K.R.); niko.poutiainen@betker.fi (N.P.); 2 Process Metallurgy Research Unit, University of Oulu, 90014 Oulu, Finland; ville-valtteri.visuri@oulu.fi (V.-V.V.); eetu.heikkinen@oulu.fi (E.-P.H.)

**Keywords:** refractories, colloidal silica, drying, calcium aluminate cement

## Abstract

Colloidal silica-bonded castables offer several advantages compared to traditional calcium aluminate cement (CAC)-bonded castables, including lower torque values during mixing, superior drying properties, and a lower CaO content. Nevertheless, information on the combination of CAC and colloidal silica is limited, and the effect of CAC additions on the drying properties of colloidal silica-bonded castables remains unknown. In this study, these drying properties were measured by rapidly heating 400 kg samples to 500 °C and assessing the resulting damage to each sample. Additionally, the physical and chemical properties of small-scale samples were analyzed to evaluate the impact of CAC addition. The analyzed properties included cold crushing strength (CCS), density, permanent linear change (PLC) and weight loss. The microstructure of the samples was investigated by FESEM and EDS. The results indicate that adding 1.5 wt.% increased the cold crushing strength at 20 °C, while lower CAC amounts had no noticeable effect. A mullite phase was observed in the sample without CAC, and correspondingly, anorthite was found in those with CAC additions. The samples exhibited significant differences in the drying tests, with the degrees of damage increasing with the CAC addition.

## 1. Introduction

Refractory castables represent a large group of materials whose application has grown significantly. Their market share has increased and, in many instances, has surpassed that of brick and shaped refractories. For many applications, they are now the most appropriate choice due to a higher performance and easier installation [1]. One application where the advantages of monolithic lining have become evident in terms of refractory consumption and higher placing efficiency is tundish backlining [2]. Despite the advancements in monolithic materials, these products still require special attention during the curing, drying, and firing stages [3]. The drying process is the most critical phase of the first heating cycle in monolithic castables, as the reduced permeability of the microstructure can lead to explosive spalling [4].

The development of high steam pressure may lead to explosive spalling and mechanical damage to the refractory lining [3]. A permeable lining will prevent steam pressure buildup, and eliminate dry-out concerns [4]. Colloidal silica-bonded castables have higher permeability levels even after polypropylene fiber addition to low and ultra-low cement castables [5]. A higher calcium aluminate cement (CAC) amount increased cold crushing strength (CCS) and decreased apparent porosity significantly, when the CAC amount ranged from 2 to 16 wt.% [6]. Theoretical aspects related to the drying process, the influence of the phase transformations derived from binder additives, and experimental techniques to assess the water removal from consolidated castable pieces were reviewed by Luz et al. [4]. Differences in the water vapor pressure inside the refractory samples were detected, resulting in a higher vapor pressure when the size of the element increased and when CAC was used as a bonding agent [7].

CAC is the most used hydraulic bonding binder due to its favorable rheological properties and green strength [8]. Green strength is supplied by the coagulation of a crystallized network through the formation of C_2_AH_8_, C_3_AH_6_, and alumina gel (AH_3_) when using CAC [9]. These phases dehydrate at intermediate temperatures up to 900 °C [10]. The drawback of adding CAC is that the presence of CaO leads to the formation of low-melting phases at high temperatures when microsilica or magnesia are used in the system [11]. Softening due to peritectic liquid formation is associated with alumina castables with microsilica and CAC in the system. The amount of peritectic liquid increases as the CAC content rises [9].

Lower torque values and shorter mixing times for colloidal silica-bonded castables were observed compared to CAC- and hydratable alumina (HA)-bonded castables [12]. The setting mechanism with microsilica is like colloidal silica, and calcium aluminate cement can be used as a coagulating agent [13]. MgO is the most suitable setting agent [14]. There is potential for improving the flowability and workability of MgO as a setting agent. MgO acts mainly as a retarder gelling agent rather than as a dispersant for the castable [15].

One of the main reasons for using colloidal silica is its high reactivity in forming mullite [1]. Colloidal silica can be absorbed to the surface of α-Al_2_O_3_, filling the packing gaps; this densification reduces the mullite formation temperature to about 1100 °C [16]. This nano-scaled expansion in mullite formation has a lower effect on the overall expansion compared to the expansions associated with CAC use [1]. A drawback of colloidal silica is that SiO_2_ will always be present in the final product, which can limit some of the applications because of chemistry and temperature [17].

There has been much research on the differences between CAC and colloidal silica [1,18,19,20]. A drawback of colloidal silica castables has been their low green strength and excessively long setting times compared to CAC-based castables [21]. Siloxane bond formation increases when CAC is added to colloidal silica-bonded castable [22]. This increased bond formation may affect the workability of the castable [1].

There is very little research about combining CAC and colloidal silica. The effects of CAC and HA addition as setting agents to colloidal silica-bonded castable have been studied. CAC + HA as a setting agent had the most positive impact on green strength. Hydratable alumina has poor drying properties and will inhibit the good drying properties of colloidal silica-bonded castables. HA-bonded castable also requires a longer mixing time and higher water content. CAC, as a setting agent, showed good green strength in the castable [21]. There are no studies concerning the drying properties of the colloidal silica-bonded castable where CAC is used as a setting agent. There are also no studies regarding the effects of different CAC additions on the physical and chemical properties of colloidal silica-bonded castables.

This article aims to investigate the optimum amount of CAC as a setting agent and its impact on the drying properties of colloidal silica-bonded castable. To this end, industrial-scale drying experiments were carried out using different CAC additions. Some of the preliminary results were presented at UNITECR 2023 [23].

## 2. Materials and Methods

Alumina castables with colloidal silica and CAC were formulated by the authors as summarized in Table 1 using the Alfred packing model with q = 0.28. Four different castable mixes were tested, with the amount of CAC being the only variable. As the amount of fine tabular alumina decreased, the CAC amount increased. The particle-size distribution was kept consistent across all mixes. Almatis supplied tabular alumina, calcined alumina, and CAC. The dead-burned magnesia used had a magnesia content of 90.5 wt.%. The dispersants employed were sodium polyacrylate, boric acid, and sodium tripolyphosphate. The consistency and fluidity of the mixtures were determined according to the ASTM 860 technical specification. Subsequently, the material was first mixed dry and again after colloidal silica and water addition, 4 min at both stages. Specimens for testing were cast under vibration.

**Figure 1:**
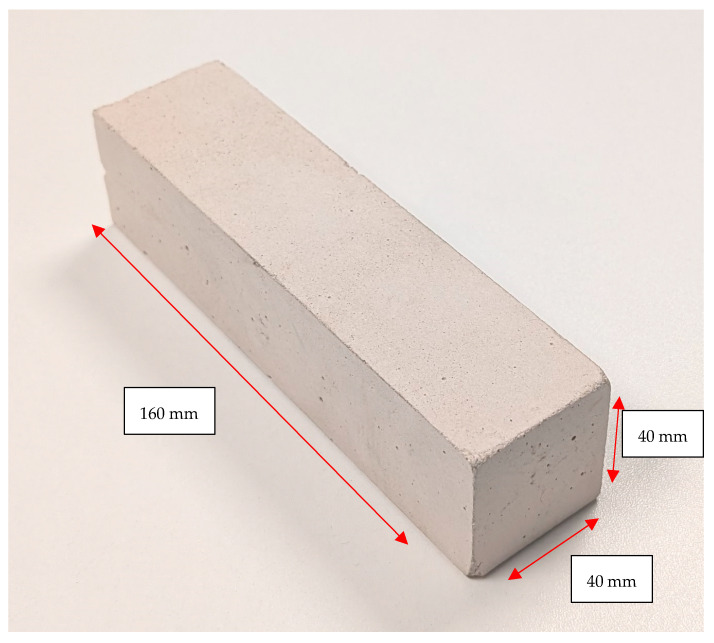
Dimensional view of the 160 mm × 40 mm × 40 mm specimen.

Specimens (160 mm × 40 mm × 40 mm) were cured first at 20 °C for 24 h and then at 110 °C for 24 h. A specimen is presented in Figure 1. Thermal treatment at 1600 °C was conducted for 2 h with Entech SF 6/17-S (Ängelholm, Sweden). The oven was heated to 1600 °C for 2 h, and cooling occurred passively. A temperature of 1600 °C was selected for the testing temperature as it is typical for the applications of this type of high-alumina castable. Cold crushing strength (CCS) was determined according to EN 933-5:2018 using a Pilot Controls 50-C92C22. Density and permanent linear changes during the thermal treatments were measured according to ISO 1927:2012. Weight loss was calculated between 110 °C and 1600 °C according to Equation (1).

Weight loss % = (W_Before firing_ − W_After firing_)/W_Before firing_ (1)

**Figure 2:**
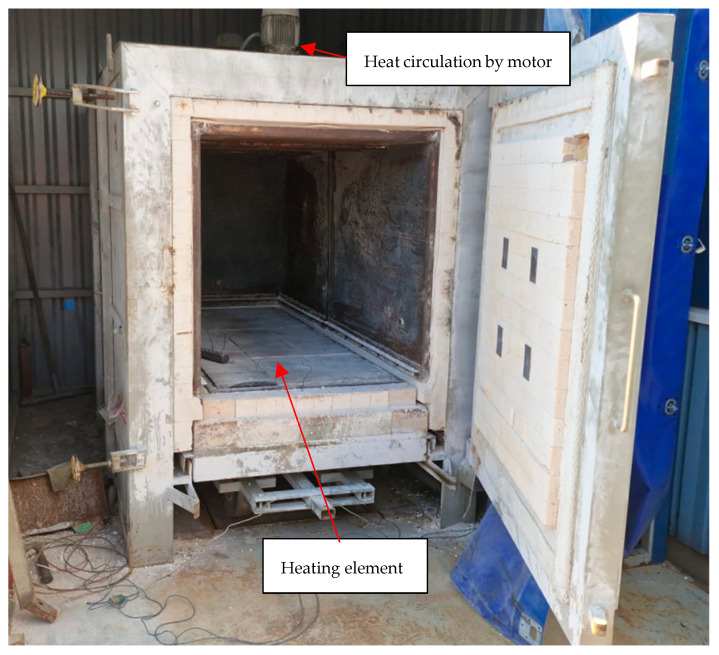
Nabertherm Mod W1500/A used for the drying tests.

The drying tests were performed using Nabertherm Mod W1500/A (Lilienthal, Germany), which is a forced convection bogie hearth oven. Electrical heating elements are inside the bogie. The air circulation ensures temperature uniformity inside the oven. The drying oven is presented in Figure 2. The heating rate was 50 °C/h from 20 °C to 500 °C. The hold time was 12 h at 500 °C, and the cooling of the oven was passive. In this test, the superior drying properties of colloidal silica-bonded castables are intended to be confirmed by the author and the limit seen where CAC causes explosive spalling and other damage to the castable. The heating rate was chosen based on the results of the simulation by Cunha et al. [24], which indicates that explosive spalling is possible when the heating rate is 50 °C/h and when the thickness is over 20 cm. Specimens for the drying test were cast into a 500 mm × 500 mm × 500 mm mold. The thermoelement was installed in the center of the specimens during casting. The thermal element for the oven was placed in the air next to the cast element. The elements were placed in the center of the oven.

Polished sections of the studied castables heat-treated at 1600 °C were investigated at the Center of Microscopy and Nanotechnology, University of Oulu, Finland, using the Field-Emission Scanning Electron Microscope (FESEM) Jeol JSM-7900F equipped (Tokyo, Japan) for energy-dispersive X-ray spectroscopy (EDS). The EDS measurements were conducted using an accelerating voltage of 15 kV and a working distance of 10 mm.

Two test series were performed: small-scale laboratory tests and industrial-scale drying tests. The industrial-scale drying tests were carried out to obtain practical results, as the surface area of the laboratory-scale samples is too large for meaningful drying test outcomes. Table 2 provides an overview of the analysis methods employed, along with the relevant test specifications.

## 3. Results and Discussion

### 3.1. Physical Tests

Various physical tests were conducted on the different castables, as shown in Table 2. The results for CCS, density, PLC, and weight loss are presented in Table 3. The addition of CAC increases the low-temperature CCS, although the density decreases. The deviation in the CCS is significant, and only 0 wt.% and 1.5 wt.% CAC exhibited overlapping results. An addition of 1.5 wt.% CAC is needed to have a clear impact on CCS at 20 °C; 1 wt.% and 1.5 wt.% CAC samples exhibit a greater CCS at 110 °C, but the results are otherwise similar to those at 20 °C. At 1600 °C, the strength values are higher with a decreasing amount of CAC, but the variation between the results remains extensive. The statistical significance was investigated by conducting unpaired *t* tests between the 0 wt.% and 1.5 wt.% results, which are presented in Table 4. Based on these results, a statistically significant difference was only found at 110 °C CCS. More samples should be tested to achieve statistically significant differences between tested samples. Mullite formation and other sintering reactions determine the high-temperature CCS, which is why the CAC does not have a significant effect on the CCS values. Density decreases as the CAC content increases. While tabular alumina has a higher density than CAC, this alone does not explain all the differences. The porosity was found to increase when the CAC addition increased, which is in keeping with the results by Gao et al. [6]. The differences in PLC and weight loss are minimal, with no clear trend for higher amounts of CAC. There were also no differences in the workability between the samples.

### 3.2. FESEM and EDS

**Figure 3:**
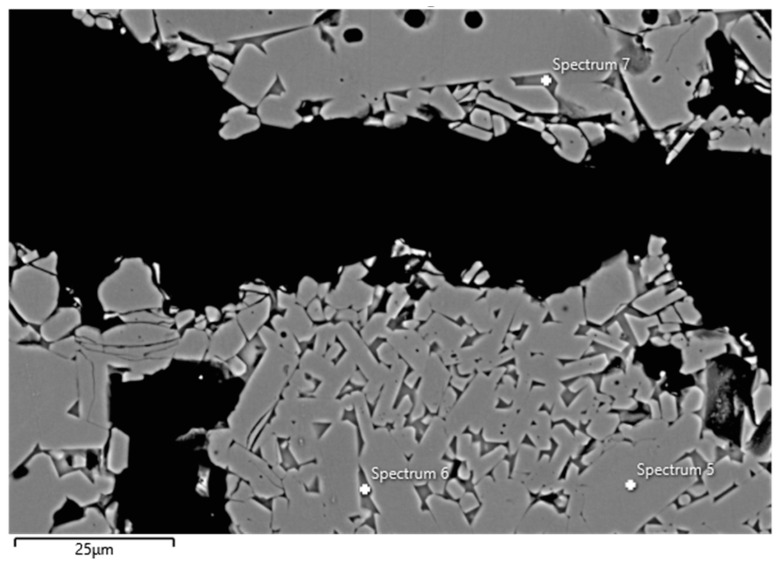
FESEM image (BSE) and EDS analyses of the 0 wt.% CAC sample; the EDS spectra are presented in Table 5.

**Figure 4:**
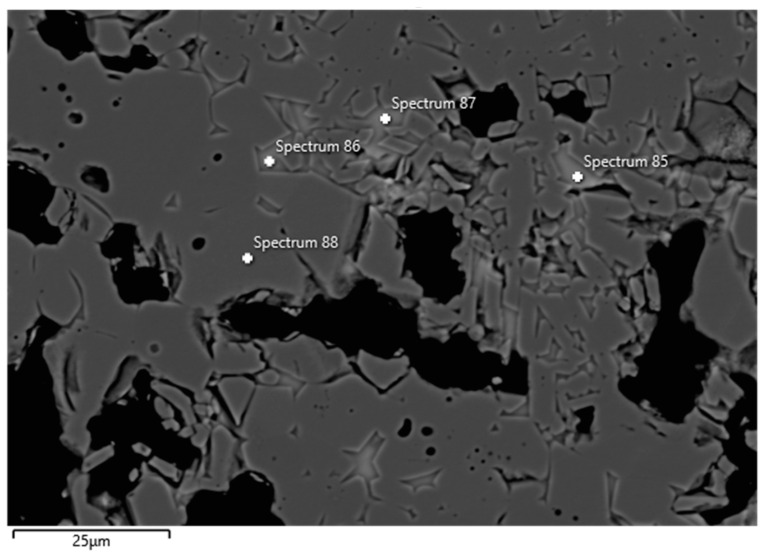
FESEM image (BSE) and EDS analyses of the 1.5 wt.% CAC sample; the EDS spectra are presented in Table 5.

Examples of FESEM images from the 0 wt.% and 1.5 wt.% CAC samples are presented in Figure 3 and Figure 4. A backscattered electron detector was used for the images. The EDS spectra and expected phases, estimated from the chemical composition of these figures, are presented in Table 5. Corundum was identified as the main phase in the aggregates, which is consistent with the composition of tabular alumina. Both samples exhibited similar structures in the matrix as presented in the FESEM images, but notable differences were observed. The 0 wt.% CAC sample had mullite as the matrix phase, with impurities of Mg and Na. This was expected when only colloidal silica was used as a binder. In contrast, the sample with a 1.5 wt.% CAC addition had anorthite as the matrix phase, with impurities from Mg and Na. The presence of anorthite was expected and aligns with the study by Myhre, who stated that peritectic liquid forms when CAC and microsilica are combined with tabular alumina as aggregates [11]. The anorthite in the 1.5% CAC sample has a peritectic melting temperature of 1512 °C, which means that there was a liquid phase when the material was heated to 1600 °C. Mullite has a melting temperature of 1880 °C, and it was found in the 0 wt.% CAC sample instead of anorthite, suggesting a better refractoriness compared to the anorthite-containing sample.

### 3.3. Drying Tests

**Figure 5:**
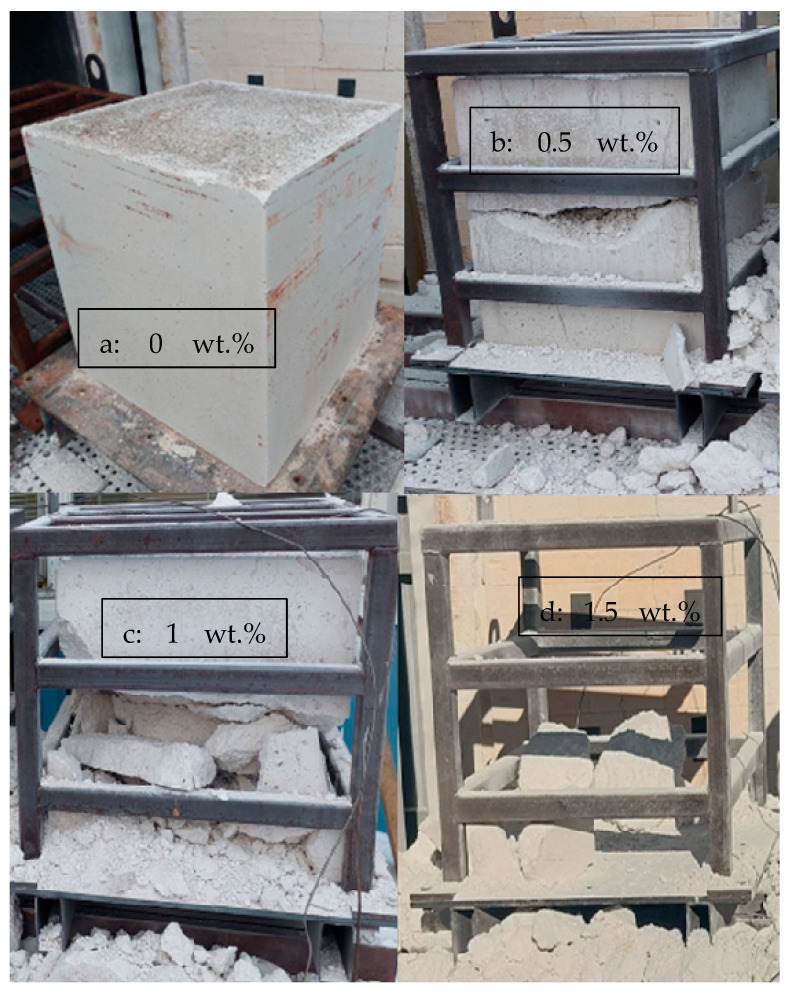
Results of the industrial-scale drying tests for 400 kg pieces with varying amounts of CAC: (**a**) 0 wt.%, (**b**) 0.5 wt.%, (**c**) 1 wt.%, and (**d**) 1.5 wt.%.

**Figure 6:**
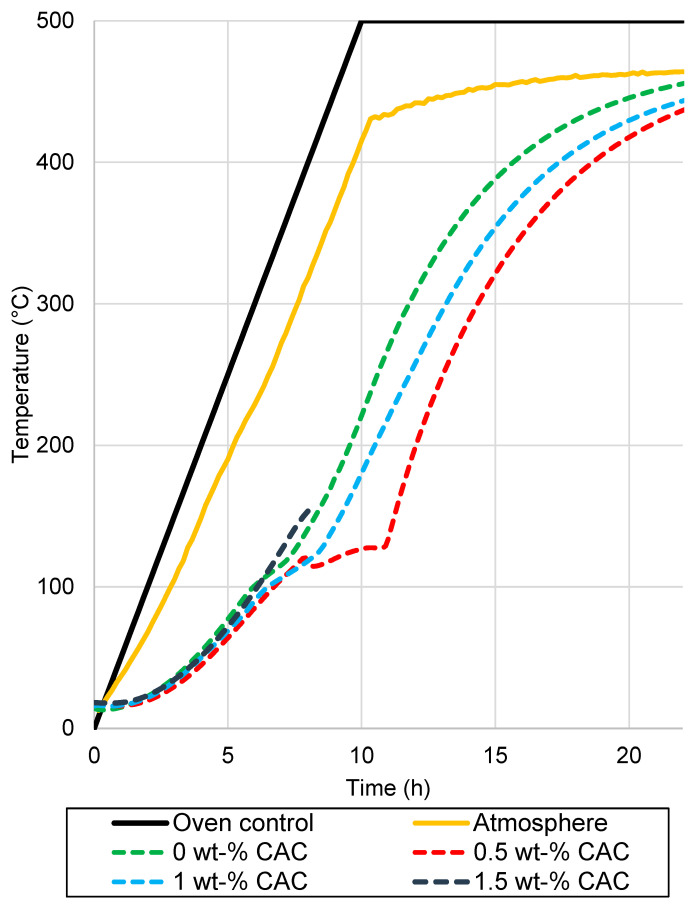
Temperature measurements from the industrial-scale drying test.

**Figure 7:**
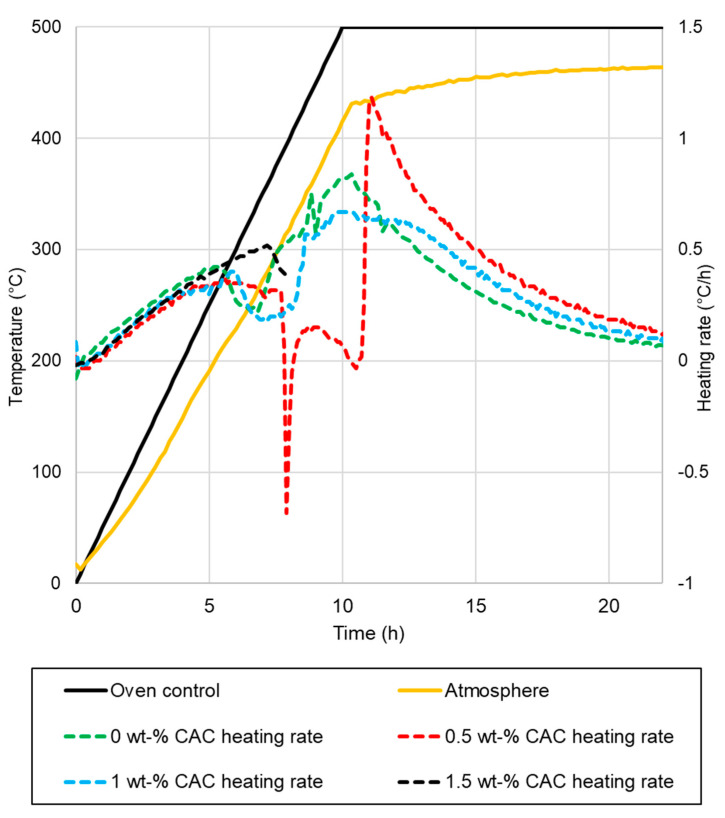
Heating rates of samples in the industrial-scale drying tests.

Drying tests were conducted for 400 kg elements in an oven, with the temperature of the pieces measured. The elements after drying are presented in Figure 5. The measured temperatures of the samples, along with the oven temperatures, are presented in Figure 6. The heating rates of the samples and temperatures of the oven are presented in Figure 7. The temperature difference between the oven control and a temperature measurement from the oven atmosphere was observed. The maximum temperature for the drying test was 460 °C, while the average heating rate was recorded at 41.11 °C/h, slightly below the target rate of 50 °C/h. The temperature curve was the same for every element, and no deviation was detected.

The results of the drying tests can be summarized as follows:

- The sample with 0% CAC (Figure 5a) remained completely intact after drying, with no observable cracks. The heating rate of the sample was steady, except between 110 °C to 140 °C, where it slowed down;
- A large crack occurred in the sample with 0.5% CAC (Figure 5b). The temperature measurement was damaged when the large crack occurred, which can be seen from the erratic heating rate data in Figure 6 between 7.5 h and 11 h. Water vapor passed by the thermocouple at that moment, disrupting the measurement. The damage occurred when the temperature in the center of the piece reached 120 °C;
- Drying the sample with 1% CAC resulted in extensive spalling and significant damage (Figure 5c). The heating rate of the sample spiked when the element’s temperature reached 120 °C. This is probably the time of the damage;
- Drying the sample with 1.5% CAC resulted in an explosion inside the sample. The damage was the most severe of all the samples, as shown in Figure 5d. The explosion occurred when the internal temperature of the element reached 150 °C.

Damage to the samples occurred when internal temperatures were between 120 °C and 150 °C. At these temperatures, pressure builds as heat drives water toward the center of the sample. This is accompanied by a decreasing heating rate in the center of the sample, as seen from the measurement data. The sudden spike in the heating rate for samples with 1% CAC occurred most probably because of damage to the samples. After the damage, there was less material to be heated, resulting in a spike in the heating rate. The 1.5% CAC sample exhibited the highest heating rate among all the samples. CAC reduces the permeability of the castable and bonds water as hydrates. More energy is directed towards heating the element and not vaporizing the water, which is an endothermic process. More drying tests are needed to obtain more reliable results on these drying properties.

Wang et al. [25] found that at the rapid dehydration stage, from 220 °C to 340 °C, the Young’s modulus decreases by approximately 50% compared to the room temperature when CAC is used as a binder. The temperature on the outer parts falls within this range, and this could mark the onset of damage. Damage occurs when the tensile strength is lower than the internal pressure.

### 3.4. Practical Implications

The values obtained for CCS indicate that it takes a 1.5 wt.% CAC addition to have a clear difference at 20 °C. Variation in the CCS for lower amounts of CAC might be problematic in practical scenarios since, in precast shape production, this is the moment when pieces are lifted to the drying oven. This can be improved by changing the material or amount of setting agent used. The other measured properties presented in Table 3 (density, PLC, weight loss) do not have the kind of differences between samples that would have practical implications.

Results from the FESEM and EBSD indicate a difference in matrix phases between the 0 wt.% CAC and 1.5 wt.% CAC samples. These phases were formed when the samples were heated to 1600 °C, which is a typical service temperature for the tested castables. The found anorthite in the 1.5 wt.% CAC sample has a melting temperature of 1570 °C, which means that there was a liquid phase when the material was heated to 1600 °C. Mullite has a melting temperature of 1880 °C, and it was found in the 0 wt.% CAC sample instead of anorthite, which indicates a better refractoriness for this sample.

A successful drying process of castables is essential when a good durability is desired in practice. The formation of cracks during drying degrades the performance of refractory linings. Results from the drying tests indicate that damage to the castable increased as the amount of CAC increased and show the benefits of colloidal silica-bonded castables and the sensitivity of CAC addition to drying properties. Furthermore, they bring out possibilities for energy-saving solutions and increased productivity when the drying time can be reduced.

The results indicate that the best performance in practice would be achieved with 0 wt.% CAC when green strength is sufficient. Except for the CCS at 20 °C, all other properties tested indicate that a 0% CAC sample would have the best performance.

### 3.5. Outlook

The results of this work indicate that to have excellent drying properties, the amount of CAC should be kept to a minimum. The main issue for these castables is a low green strength, which should be addressed in future development. A new setting agent may be needed to enhance the gelation effect on the colloidal silica. On the other hand, CAC addition improves CCS at lower temperatures. While the drying properties are degraded, these can be mitigated by adjusting the drying curve. Depending on the final application, either approach of developing these colloidal silica-bonded castables could be viable.

## 4. Conclusions

Colloidal silica-bonded castables offer many benefits compared to traditional CAC-bonded castables, including superior drying properties and a lower amount of CaO in the material. This study aimed to provide new insights into the effects of CAC addition on the properties of colloidal silica-bonded castables. The drying properties were assessed by heating 400 kg samples rapidly and determining the resulting damage to each sample. Additionally, the physical and chemical properties of small-scale samples were analyzed to evaluate the effects of CAC addition.

The results indicate that a 1.5% CAC addition provides a higher CCS at 20 °C, while lower amounts of CAC did not have a noticeable impact. Differences in the matrix were observed between the 0 wt.% and 1.5 wt.% CAC samples. Mullite was present in the 0 wt.% CAC sample, whereas anorthite was found in the matrix of the 1.5 wt.% CAC sample. Both phases contained impurities in Mg and Na. The samples exhibited significant differences in the drying tests: the damage increased with the increased addition of CAC. No damage was observed in the 0% CAC sample, while explosive spalling was detected in the 1.5% CAC sample. These results highlight the advantages of colloidal silica-bonded castables and the sensitivity of drying properties to CAC addition.

Based on the results of this work, it is not possible to provide a definite answer regarding the optimum amount of CAC addition. It appears that while a 1.5% CAC addition is needed to have a clear impact on the 20 °C CCS, this comes at the expense of the material’s drying properties. Further research is necessary to quantify this effect in more detail. Countermeasures to address weakened drying properties can be developed by adjusting the drying curve.

## Figures and Tables

**Table 1:** Castable formulation (wt.%).

Raw Materials	0 wt.% CAC	0.5 wt.% CAC	1 wt.% CAC	1.5 wt.% CAC
Tabular alumina 0–10 mm (Almatis, Germany)	84.8	84.3	83.8	83.3
Calcined alumina (Almatis, Germany)	12.9	12.9	12.9	12.9
Dead-burned MgO (China)	0.2	0.2	0.2	0.2
CAC 70% (CA-14 Almatis, The Netherlands)	0	0.5	1	1.5
Colloidal silica suspension (40 wt.% solids)	2% solids	2% solids	2% solids	2% solids
Dispersant	0.1	0.1	0.1	0.1
Water (wt.%)	3.5%	3.5%	3.5%	3.5%

**Table 2:** Overview of the analysis methods employed.

Test Series	Laboratory-Scale	Industrial-Scale	Note
Cold Crushing Strength	X		EN 933-5:2018
Density	X		ISO 1927:2012
Permanent Linear Change	X		ISO 1927:2012
Weight Loss	X		Equation (1)
FESEM Analysis	X		Thermal-treated at 1600 °C
Casting Properties	X	X	ASTM 860
Drying Properties		X	0.5 m × 0.5 m × 0.5 m cubic

**Table 3:** Properties measured from the castables.

		0 wt.% CAC			0.5 wt.% CAC			1 wt.% CAC			1.5 wt.% CAC		
T (°C)	Property	Value	Avg.	St. Dev.	Value	Avg.	St. Dev.	Value	Avg.	St. Dev.	Value	Avg.	St. Dev.
20 °C	CCS (MPa)	12.54	22.08	8.30	12.38	22.72	9.04	19.37	33.87	2.11	30.92	33.23	2.47
		26.12			26.65			32.37			32.93		
		27.59			29.14			35.36			35.84		
	Density (kg/m^3^)	3.14	3.10	0.04	3.1	3.08	0.03	3.11	3.08	0.03	3.01	3.05	0.04
		3.09			3.05			3.06			3.07		
		3.06			3.08			3.08			3.08		
110 °C	CCS (MPa)	48.01	47.00	1.63	33.65	41.61	7.45	49.00	56.68	7.49	67.75	63.13	5.49
		47.87			42.77			57.07			64.57		
		45.12			48.41			63.96			57.06		
	Density (kg/m^3^)	3.01	3.05	0.04	3.05	3.04	0.01	3.03	3.04	0.02	3.01	3.02	0.01
		3.08			3.03			3.02			3.02		
		3.06			3.03			3.06			3.02		
1600 °C	CCS (MPa)	141.98	180.99	38.40	111.69	157.02	40.34	123.09	167.13	38.32	152.59	171.94	43.40
		218.74			170.40			185.47			141.58		
		182.26			188.98			192.84			221.65		
	Density (kg/m^3^)	3.11	3.15	0.04	3.12	3.15	0.03	3.11	3.14	0.04	3.09	3.12	0.04
		3.19			3.16			3.12			3.11		
		3.14			3.18			3.18			3.17		
	PLC (%)	−1.43	−1.30	0.13	−1.04	−1.50	0.40	−1.31	−1.43	0.10	−1.33	−1.45	0.23
		−1.30			−1.77			−1.50			−1.31		
		−1.17			−1.69			−1.47			−1.71		
	Weight loss (%)	0.30	0.30	0.00	0.20	0.27	0.06	0.40	0.33	0.06	0.30	0.37	0.06
		0.30			0.30			0.30			0.40		
		0.30			0.30			0.30			0.40		

**Table 4:** Results of unpaired *t* test between 0 wt.% CAC and 1.5 wt.% CAC samples.

		0 wt.% CAC		1.5 wt.% CAC			
T (°C)	Property	Avg.	St. Dev.	Avg.	St. Dev.	Mean Difference	*p*-Value
20 °C	CCS (MPa)	22.08	8.3	22.72	9.04	0.64	0.09
	Density (kg/m^3^)	3.10	0.04	3.08	0.03	0.02	0.25
110 °C	CCS (MPa)	47.00	1.63	41.61	7.45	5.39	0.01
	Density (kg/m^3^)	3.05	0.04	3.04	0.01	0.01	0.19
1600 °C	CCS (MPa)	180.99	38.40	157.02	40.34	23.97	0.80
	Density (kg/m^3^)	3.15	0.04	3.15	0.03	0.00	0.52
	PLC (%)	−1.30	0.13	−1.5	0.40	0.20	0.37
	Weight loss (%)	0.30	0.00	0.27	0.06	0.03	0.12

**Table 5:** EDS spectra from Figure 3 and Figure 4.

Spectrum (wt.%)	5	6	7	85	86	87	88
O	46.07	44.21	45.57	44.92	45.21	45.19	45.27
Al	53.93	29.82	21.14	16.29	16.64	16.69	54.73
Si		19.89	23.9	21.57	21.23	20.91	
Ca		0.49	0.72	9.26	8.85	9.16	
Na		3.38	4.71	4.28	4.28	4.3	
Mg		1.86	3.47	3.44	3.41	3.51	
Expected phase ^1^	Corundum	Mullite	Mullite	Anorthite	Anorthite	Anorthite	Corundum

^1^ Phases are estimated based on chemical composition of spectra.

## Data Availability

The original contributions presented in the study are included in the article, further inquiries can be directed to the corresponding author.

## Nomenclature

AH_3_	Al(OH)_3_-Gibbsite-Bayerite
BSE	Backscattered electron
CAC	Calcium aluminate cement
C_2_AH_8_	Ca_2_Al_2_O_13_H_16_—Dicalcium aluminate octahydrate
C_3_AH_6_	Ca_3_[Al(OH)_6_]_2_—Katoite-Hydrogarnet
CaO	Calcium oxide
CCS	Cold crushing strength
EDS	Energy-dispersive X-ray spectroscopy
FESEM	Field-Emission Scanning Electron Microscope
HA	Hydratable alumina
MgO	Magnesium oxide
PLC	Permanent linear change
SiO_2_	Silicon dioxide
α-Al_2_O_3_	Alpha aluminium oxide

## References

[1] Nouri-Khezrabad M., Braulio M.A.L., Pandolfelli V.C., Golestani-Fard F., Rezaie H.R. (2013). Nano-Bonded Refractory Castables. Ceram. Int..

[2] Da Luz A.P., Braulio M.D.A.L., Pandolfelli V.C. (2015). Refractory Castable Engineering.

[3] Luz A.P., Moreira M.H., Braulio M.A.L., Parr C., Pandolfelli V.C. (2021). Drying Behavior of Dense Refractory Ceramic Castables. Part 1—General Aspects and Experimental Techniques Used to Assess Water Removal. Ceram. Int..

[4] Luz A.P., Moreira M.H., Salomão R., Braulio M.A.L., Pandolfelli V.C. (2022). Drying Behavior of Dense Refractory Castables. Part 2—Drying Agents and Design of Heating Schedules. Ceram. Int..

[5] Parr C., Aluminates I., Wohrmeyer C., Touzo B., Bell D. (2001). Technical Paper; The Role of Calcium Aluminate Cement During the Installation and Dry Out of High Purity Alumina Castables.

[6] Gao S., Zhang P., Li N., Zhang J., Luan J., Ye G., Liao G. (2020). Effect of CAC Content on the Strength of Castables at Temperatures between 300 and 1000 °C. Ceram. Int..

[7] Moreira M.H., Peng H., Pont S.D., Pandolfelli V.C. (2023). Can a Laboratory Experiment Express the Size Effect on the Drying of Refractory Castables?. Ceram. Int..

[8] Bayoumi I.M.I., Ewais E.M.M., El-Amir A.A.M. (2021). Rheology of Refractory Concrete: An Article Review. Bol. Soc. Esp. Ceram. Vidr..

[9] Liu H., Chen W., Pan R., Shan Z., Qiao A., Drewitt J.W.E., Hennet L., Jahn S., Langstaff D.P., Chass G.A. (2020). From Molten Calcium Aluminates through Phase Transitions to Cement Phases. Adv. Sci..

[10] Lee W.E., Vieira W., Zhang S., Ahari K.G., Sarpoolaky H., Parr C. (2001). Castable Refractory Concretes. Int. Mater. Rev..

[11] Myhre B. (2008). Let´s Make a Mullite Matrix!. Refract. Appl. News.

[12] Salomão R. (2006). Colloidal Silica as a Nanostructured Binder for Refractory Castables. Refract. Appl. News.

[13] Peng H., Myhre B. (2017). Improvements in drying behaviour and explosion resistance of microsilica-gel bonded no-cement castables. Refract. WorldForum.

[14] Dos Anjos R.D., Ismael M.R., De Oliveira I.R., Pandolfelli V.C. (2008). Workability and Setting Parameters Evaluation of Colloidal Silica Bonded Refractory Suspensions. Ceram. Int..

[15] Nouri-Khezrabad M., Luz A.P., Salvini V.R., Golestani-Fard F., Rezaie H.R., Pandolfelli V.C. (2015). Developing Nano-Bonded Refractory Castables with Enhanced Green Mechanical Properties. Ceram. Int..

[16] Xiong J., Peng Y., Xie D., Mao X. (2012). The Characteristics of Silica-Sol Combining Refractories. Adv. Mater. Res..

[17] Braulio M.A.L., Tontrup C., Medeiros J., Pandolfelli V.C. (2011). Colloidal Alumina as a Novel Castable Bonding System. Refract. Worldforum.

[18] Burgos-Montes O., Álvarez M., Restrepo E., De Aza A.H., Pena P., Baudín C. Influence of the silica gel technology on the high temperature mechanical behaviour of alumina castables. Proceedings of the UNITECR 2017—15th Biennial Worldwide Congress.

[19] Jia Q., Zhang J., Zhou Y., Jia G., Liu X. (2018). Effect of Microsilica Addition on the Properties of Colloidal Silica Bonded Bauxite-Andalusite Based Castables. Ceram. Int..

[20] Qiu W., Ruan G., Zhang Z. (2018). Properties of Silica Sol Bonded Corundum-Spinel Castables for Steel Ladles. Int. J. Appl. Ceram. Technol..

[21] Nouri-Khezrabad M., Luz A.P., Salvini V.R., Golestani-Fard F., Rezaie H.R., Pandolfelli V.C. (2015). PaPers Novel Engineering Route to Improve the Green Mechanical Properties of Nano-Bonded Refractory Castables. Refract. WorldForum.

[22] Yaghoubi H., Sarpoolaky H., Golestanifard F., Souri A. (2012). Influence of nano silica on properties and microstructure of high alumina ultra-low cement refractory castables. J. Mater. Sci. Eng..

[23] Piippo A., Ruotanen K., Visuri V.-V., Poutiainen N., Heikkinen E.-P. Observations on the strength and drying performance of SolCast castables. Proceedings of the Unitecr 2023.

[24] Cunha T.M., Moreira M.H., Santos M.F., Angélico R.A., Pandolfelli V.C. (2023). A Simple Methodology Based on Numerical Analysis for the Drying Curve Design of a Castable Lined Steel Ladle. Open Ceram..

[25] Wang Y., Li X., Zhu B., Chen P. (2016). Microstructure Evolution during the Heating Process and Its Effect on the Elastic Properties of CAC-Bonded Alumina Castables. Ceram. Int..

